# Vocal individuality in drumming in great spotted woodpecker—A biological perspective and implications for conservation

**DOI:** 10.1371/journal.pone.0191716

**Published:** 2018-02-07

**Authors:** Michał Budka, Krzysztof Deoniziak, Tomasz Tumiel, Joanna Teresa Woźna

**Affiliations:** 1 Department of Behavioural Ecology, Faculty of Biology, Adam Mickiewicz University, Poznan, Poland; 2 Laboratory of Insect Evolutionary Biology and Ecology, Institute of Biology, University of Bialystok, Bialystok, Poland; 3 Wildlife Society “Dubelt”, Juszkowy Gród, Poland; 4 Institute of Zoology, Poznań University of Life Sciences, Poznań, Poland; Hungarian Academy of Sciences, HUNGARY

## Abstract

Animals—including conservation biologists—use acoustic signals to recognise and track individuals. The majority of research on this phenomenon has focused on sounds generated by vocal organs (e.g., larynx or syrinx). However, animals also produce sounds using other parts of the body, such as the wings, tail, legs, or bill. In this study we focused on non-syrinx vocalisation of the great spotted woodpecker, called drumming. Drumming consists of strokes of a bill on a tree in short, repeated series, and is performed by both males and females to attract mates and deter rivals. Here, we considered whether the great spotted woodpecker’s drumming patterns are sex-specific and whether they enable individual identification. We recorded drumming of 41 great spotted woodpeckers (26 males, 9 females, 6 unsexed). An automatic method was used to measure the intervals between succeeding strokes and to count strokes within a drumming roll. The temporal parameters of drumming that were analysed here had lower within- than between-individual coefficients of variation. Discriminant function analyses correctly assigned 70–88% of rolls to the originating individual, but this depended on whether all individuals were analysed together or split into males and females. We found slight, but significant, differences between males and females in the length of intervals between strokes—males drummed faster than females—but no difference in the number of strokes within a roll. Our study revealed that temporal patterns of drumming in the great spotted woodpecker cannot be used for unambiguous sex determination. Instead, discrimination among individuals may be possible based on the intervals between strokes and the number of strokes within a roll. Therefore, it is possible that differences in the temporal parameters of drumming may be used by birds to identify each other, as well as by researchers to aid in census and monitoring tasks.

## Introduction

Vocal individuality is usually considered in one of two contexts: practical—when biologists and ecologists utilise acoustic features to identify individuals [1], and biological—when animals use unique characteristics of vocalisation to identify each other [2–3]. Regardless of the context, two terms should be clearly distinguished when vocal individuality is considered: discrimination and identification [1]. Discrimination requires that two individuals differ enough at one point in time to be separated. Simply put, in at least two individuals, variation of a particular feature of the call should not overlap. Thus, discrimination addresses only the question of whether observations are being made on different individuals or not necessarily on different (i.e. different or the same). This is especially important when researchers count individuals living within a population (to avoid double-counting of the same individual) [1], or in field experiments in which only a single observation of a given individual can be used in order to avoid pseudoreplication [4]. In a biological context, discrimination means that animals are able to classify a particular individual as belonging to a specific class or group. For example, territorial skylarks (*Alauda arvensis*) discriminate individuals as belonging to one of two categories: their familiar neighbours or unfamiliar stranger birds [3]. Ravens (*Corvus corax*), on the other hand, are able to discriminate familiar group members from unfamiliar individuals even after a long period of separation [5].

Identification, instead, requires that a feature of the vocalisation be unique, individually specific, and constant over time, thus enabling the recognition of a particular individual across time and space with 100% confidence [6]. In this case, the within-individual variation of an individually specific feature does not overlap with that of other individuals. In conservation biology, identification aids in tracking the movements of individuals and their life histories [1]. From a biological perspective, identification is very important both for senders and receivers, since it enables signalling and the perception of identity by animals. For example, female zebra finches (*Taeniopygia guttata castanotis*) are able to identify their mates [7] and king penguin (*Aptenodytes patagonicus*) chicks can find their parents in colonies comprising several thousands of birds [2]. From another point of view, vocal identification is also considered to be the probability that two calls belong to the same individual [8]. In such approach researchers provide for find two individuals who cannot be distinguished. However, this probability is extremely low, like it is unlikely to find two individuals with undistinguishable fingerprint in humans.

Vocal individuality has been described in various animal species, including fish [9], amphibians [10], and mammals [11]. However, acoustic discrimination or identification has been most intensively studied in birds, in which individually specific calls or songs have been found, for example, in European nightjars (*Caprimulgus europaeus*) [12], woodcocks (*Scolopax rusticola*) [13], European eagle owls (*Bubo bubo*) [14], corncrakes (*Crex crex*) [15], and common cuckoos (*Cuculus canorus)* [16]. Certain characteristics of the call or song in all the above-mentioned species have lower within- than between-individual variation, and in some of them call or song characteristics are constant over an individual’s lifetime. In addition, vocalisations in these species are generated by a vocal organ—the syrinx. However, some birds also produce non-syrinx vocalisations, which are generated by other parts of the body. Storks, for example, produce acoustic signals called “clatter” by rattling their mandibles together. In the oriental white stork (*Ciconia boyciana*), the clatter is sex-specific and enables discrimination between males and females [17]. Males of the common snipe (*Gallinago gallinago*) produce a “drumming” sound with their outer tail feathers during their mating dives [18]. Woodpeckers likewise produce “drumming”, which in this case is generated by a rapid, repetitive series of pecking by the bill on a substrate [19]. The acoustic structure of woodpecker drumming varies among species [20–21].

The function of non-syrinx vocalisations is similar to those produced by a syrinx: mate attraction and territorial announcement [17–19]. Regardless of whether the signal is generated by vibration of the membranes in a syrinx or by another part of a bird’s body, the physical characteristics of an acoustic signal should depend on the size and shape of the sound source. Therefore, the anatomy of a sound production organ should limit the acoustic properties of a signal produced by a particular individual [22–24]. Unfortunately, data on non-syrinx vocalisations are scarce, and in many cases, we know little about the mechanism of sound production, within- and between-individual variation, and even the biological function of non-syrinx vocalisations.

In our study we focused on vocal individuality in non-syrinx vocalisations of the great spotted woodpecker (*Dendrocopos major*). The great spotted woodpecker is the most common and best-known woodpecker species in the Western Palearctic. It inhabits various types of forests, parks, and groves [25], and in most of the species’ range, the birds are year-round residents. During the breeding season, males and females form socially monogamous pairs, then settle and defend their territory [26]. Both sexes produce non-syrinx vocalisations, called drumming [25]. The drumming is generated by strokes of the bill on a substrate in short, repeated series. As a substrate, birds usually use a branch or a trunk of a tree, but also anthropogenic substrates like wooden housing supports, lampposts, telephone poles, or steel tiles [25]. To the best of our knowledge, no study has yet described in detail the acoustic characteristics of great spotted woodpecker drumming in the context of sex discrimination and individual identification. Indeed, to date, the only mention of drumming as it relates to discrimination and identification was made by Zabka [27], who suggested that drumming is probably important in these biological contexts.

In this study we considered (1) whether drumming in the great spotted woodpecker is sex-specific, and (2) whether temporal patterns of drumming enable discrimination among or identification of individuals. Additionally, we discuss (3) the potential of non-syrinx vocalisation to serve as a tool in the census and monitoring of our study species and of other, less numerous woodpeckers, and (4) the potential biological functions of drumming.

## Methods

### Ethics statement

The study was conducted in full compliance with the current laws of the Poland. We recorded birds on places with unrestricted public accesses, therefore no specific permissions were required for these locations. The study was purely observational, non-invasive and done on wild animals, therefore no special permits were required.

### Study sites and drumming recording

We recorded the drumming of great spotted woodpeckers in three regions in Poland: Greater Poland (52.47° N 16.99° E; 10 individuals: 7 males, 2 females, 1 unsexed), Masovia (52.31° N 20.90° E; 9 individuals: 7 males, 0 females, 2 unsexed), and Podlasie (53.02° N 23.51° E; 22 individuals: 11 males, 7 females, 3 unsexed). The distance between locations ranged from 190 km (Masovia—Podlasie) to 440 km (Greater Poland—Podlasie). However, the distances between individuals recorded in the same location were also large, reaching up to 95 km. In Poland, the great spotted woodpecker is widespread in all type of wooded habitats, and its population is continuous, without any isolating barriers [28]. Therefore, we treated all recordings that we collected as belonging to a single population.

Recordings were taken during three breeding seasons, in March-May of 2014–2016. Birds were recorded using either a Marantz PMD661 recorder connected to a Sennheiser ME 67 directional microphone and K6 power module, a Marantz PMD670 recorder connected to a Telinga Pro 6 microphone mounted on a Telinga Universal parabola, or an Olympus LS-100 recorder connected to a Sennheiser ME66 directional microphone and K6P power module. All recordings had the same digital quality– 48 kHz/16 bit sample rate. We did not use playback to stimulate woodpeckers to drum. When possible, we noted the sex of the drumming bird. To avoid multiple recordings of the same individual, we recorded birds in a given location only one day, and only if we were certain that we recorded different individuals. Therefore, the probability that the same bird was recorded two times was near zero.

### Drumming analysis

**Fig 1:**
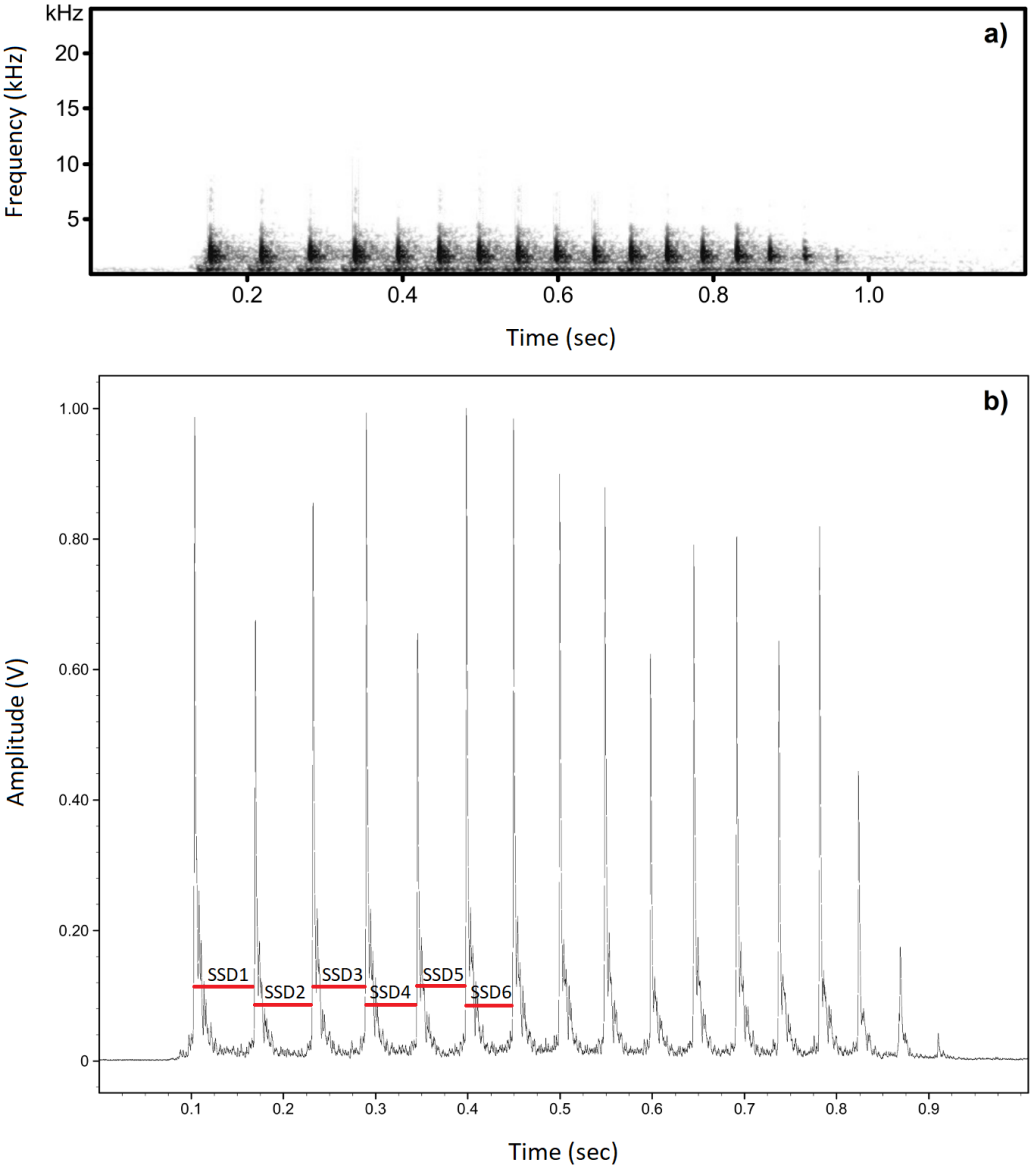
Spectrogram of great spotted woodpecker drumming. (a) Spectrogram represents one roll of drumming. (b) Pulse Train Analysis window with visible strokes within drumming roll (first six stroke-to-stroke durations are indicated). Spectrogram settings: FFT length = 512; Frame size = 75%, Window = Hamming.

Drumming was analysed using Avisoft SASLab Pro software v 5.2.10 (Avisoft Bioacoustics, Germany). First, we removed background noise from each recording using a high-pass, time-domain filter (FIR) with a cutoff of 0.5 kHz. Then, we measured the time between succeeding strokes (stroke-to-stroke duration, or SSD) and counted the number of strokes in each roll (Fig 1, S1 File). We applied an automatic method to our measurements using the Pulse Train Analysis function with the following settings: hysteresis = 15 dB, start/end threshold = -10 dB, threshold = 0.03 V, and time constant = 1 ms. However, in a few recordings we manipulated the values of hysteresis, start/end threshold, and threshold in order to correctly detect and measure all strokes. These manipulations were necessary mainly because of the varying amplitude of recorded sounds or because of variations in the general recording quality, and did not influence the measured temporal parameters of drumming.

When a great spotted woodpecker drums, the sound is generated by the bill, whose strokes are propelled by neck muscles [25]. Bill size is constant over an adult bird’s life, while the characteristic pattern of the neck muscles probably determines the specific temporal structure of drumming [29]. Therefore, the temporal parameters of drumming measured here—SSDs—should be constrained by anatomy and thus be individually specific. Instead, the second component of drumming—the substrate—is variable both within- and between-individuals [25]. The resonant properties of a substrate may strongly affect the spectral drumming characteristics (i.e. energy distribution in frequency range, bandwidth, amplitude, loudness) [30], but are unlikely to influence temporal characteristics [27]. Therefore, in our analyses, we focused only on temporal characteristics of drumming and excluded spectral characteristics.

### Statistical analyses

We examined whether acoustic parameters of great spotted woodpecker drumming enable sex discrimination and individual identification. Because recordings were collected in three different locations, we first examined geographic differences in drumming characteristics. To do this, we calculated the average values of drumming parameters for each individual and then compared them using t-tests.

To determine which drumming characteristics could be useful for individual identification, we first calculated within-individual (CV_i_) and between-individual (CV_b_) coefficients of variation for each parameter. We used the formula [CV = 100 × (1 + 1 / (4 × n)) × SD / mean], where *n* is sample size [31]. Then, we calculated the potential for identity coding (PIC) for each analysed drumming parameter as the ratio of CV_b_ / CV_i_ [32]. PIC values greater than 1 indicated that the within-individual variation was lower than between-individual variation for a particular drumming characteristic, which meant that it could potentially be used for individual identification. In our study population we found that the lowest number of strokes observed in a roll was 6. The parameters included in our final analyses were therefore the number of strokes within a roll (NS), the first five SSDs (SSD1-SSD5), and the minimum SSD (SSD_min_) within a roll.

For the classification of drumming, we conducted three separate stepwise discriminant function analyses (DFAs). Predictors were selected by using Wilks’ lambda criterion. The probability of F was used as a criterion of entering or removing a variable from a model (p-to-enter = 0.05; p-to-remove = 0.10). In the first analysis we considered the drummings of all 41 individuals together, while in the second we considered only the 26 males, and in the third only the 9 females. In the DFAs we used as initial predictors: NS, SSD1-SSD5, and SSD_min_. Some of our initial predictors were highly correlated (Table 1), therefore we excluded from the analyses highly mutually correlated predictors (when r > 0.80). Additionally, we checked a Variance Inflation Factor (VIF) for collinearity diagnostic for selected predictors. In these three DFAs, prior probabilities were computed from group sizes. We used a within-group covariance matrix and applied a ‘leave-one-out classification’ as a cross-validation method. Additionally, to examine how the rate of correct classification changed when different numbers of SSDs were used in the models, we performed a series of DFAs with different numbers of predictors. Our null model contained only one predictor, that with the highest PIC value. We then added predictors one at a time in order of decreasing PIC value, until the model finally contained all 12 predictors. The final number of DFA models tested was 12. In these analyses we considered only 33 individuals (454 rolls; from 5 to 33 rolls per individual) for which we observed at least 11 strokes in each roll.

**Table 1:** Correlation matrix between initial predictors used in discriminant function analysis to classify individuals.

	**NS**	**SSD1**	**SSD2**	**SSD3**	**SSD4**	**SSD5**
**SSD1**	0.009					
**SSD2**	0.190*	0.793*				
**SSD3**	0.212*	0.712*	0.915*			
**SSD4**	0.268*	0.613*	0.872*	0.918*		
**SSD5**	0.311*	0.513*	0.786*	0.899*	0.917*	
**SSD_min_**	-0.366*	0.219*	0.349*	0.403*	0.412*	0.422*

Results based on 609 rolls belonging to 41 individuals. Pearson’s r coefficients are given.

*—correlation is significant at the 0.01 level.

Mutual correlations with r > 0.80 are bold. SSD3 and SSD4 were excluded from DFA (r > 0.8)

Differences in drumming characteristics between males and females were analysed also with stepwise DFA, in which we used sex as grouping variable and NS, SSD1-SSD5, and SSD_min_ as initial predictors (average values for each individual). Some of our initial predictors used in this analysis were highly correlated (Table 2), therefore, we excluded them from further DFA (when r > 0.80). Additionally, we also checked a VIF for collinearity diagnostic between predictors included in the final model. Prior probability was computed from group sizes. We used a within-group covariance matrix and applied a ‘leave-one-out classification’.

**Table 2:** Correlation matrix between initial predictors used in discriminant function analysis to sex discrimination.

	**NS**	**SSD1**	**SSD2**	**SSD3**	**SSD4**	**SSD5**
**SSD1**	-0.04					
**SSD2**	0.147	0.832**				
**SSD3**	0.179	0.710**	0.962**			
**SSD4**	0.216	0.599**	0.914**	0.979**		
**SSD5**	0.282	0.499**	0.834**	0.928**	0.973**	
**SSD_min_**	-0.296	0.178	0.416*	0.487**	0.541**	0.556**

Results based on average values of drumming characteristics of 35 individuals. Pearson’s r coefficients are given.

**—correlation is significant at the 0.01 level;

*—correlation is significant at the 0.05 level.

Mutual correlations with r > 0.80 are bold. SSD3 and SSD4 were excluded from DFA (r > 0.8)

Statistical analyses were performed in IBM SPSS Statistics 23. The normality of distributions was checked by Kolmogorov-Smirnov test. All p-values are two-tailed.

## Results

**Fig 2:**
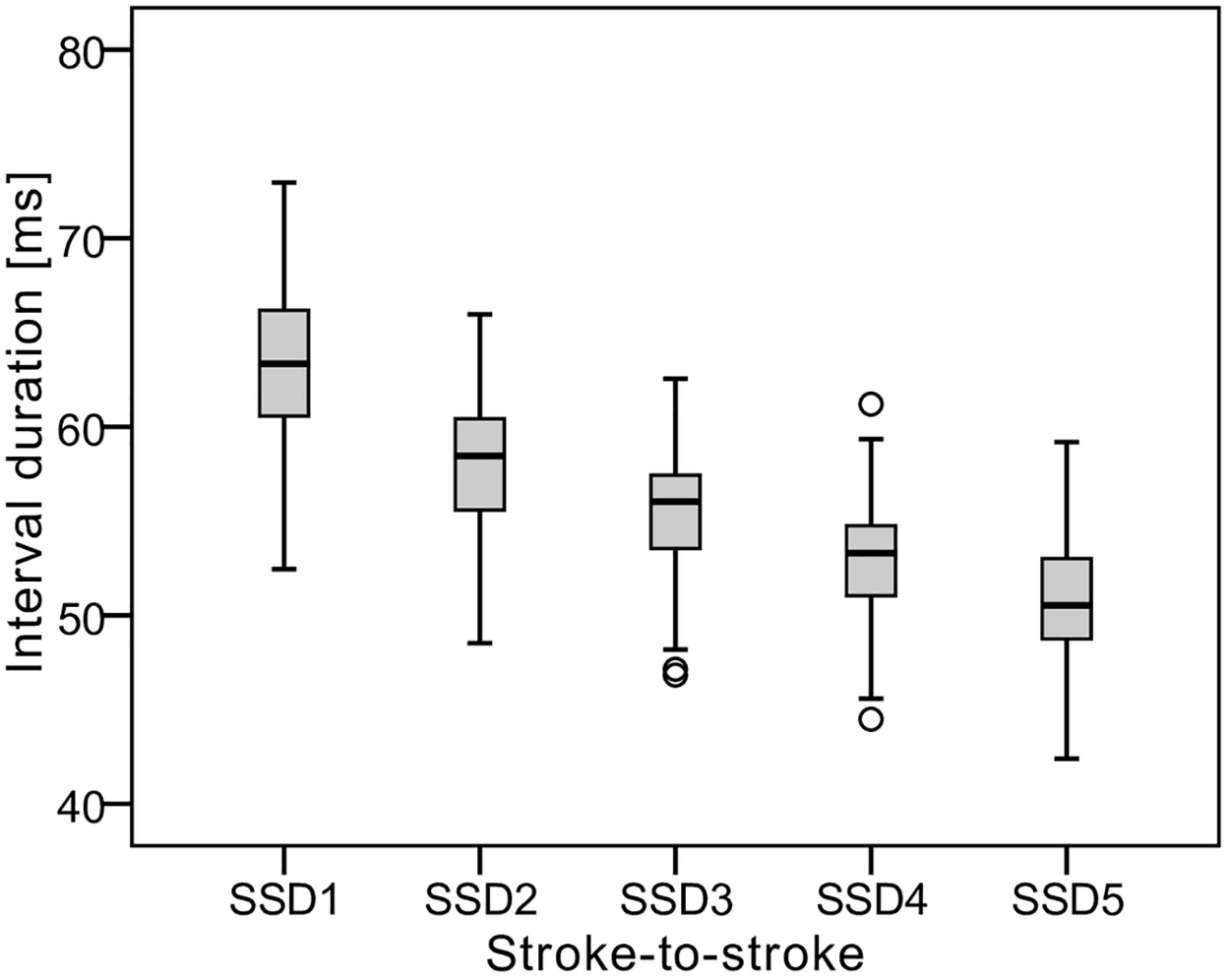
Interval duration between succeeding strokes within a roll. First five stroke-to-stroke durations (SSD1-SSD5) and minimal stroke-to-stroke duration within a roll (SSDmin) are given. Figure based on average values of 41 drumming individuals.

We recorded 609 great spotted woodpecker rolls, belonging to 41 individuals: 26 males, 9 females, and 6 individuals for which we could not determine the sex (S1 Dataset). From each individual we recorded on average 15± 9.2 rolls (range from 5 to 41). Each roll contained on average 12± 2.8 strokes (range from 6 to 20). The time between succeeding strokes significantly decreased as a roll progressed (GLM REP: Pillai’s Trace value = 0.940; F_4,37_ = 143.78; p < 0.001) (Fig 2). Therefore, drumming in the great spotted woodpecker should be classified as accelerative. We did not find significant differences in drumming parameters among the three locations in which recordings were collected (t-test; NS: t_19_ = 1.804; SSD1: t_19_ = -1.846; SSD2: t_19_ = -1.119; SSD3: t_19_ = -0.722; SSD4: t_19_ = -0.101; SSD5: t_19_ = 0.750; SSD_min_: t_19_ = 0.393; in all cases p > 0.05).

### Sex discrimination

We compared the average number of strokes, minimum SSD, and first five SSDs between males and females. We found that males had slightly but significantly shorter SSDs, but the range overlapped between males and females (Table 3). In a stepwise DFA, only one predictor—SSD2—was selected into the model. DFA correctly classified sex of individuals based on SSD2 in 82.9% of cases (82.9% in leave-one-out classification) (Wilks’ lambda = 0.798, χ^2^ = 7.546, df = 1, p = 0.006). We also conducted second DFA, in which we included two additional predictors (NS and SSD_min_) which correlated less than 0.8 with SSD2 (VIF < 1.21) and entered them together into the model. This DFA model did not improve correct classification rate, since also 82.9% of rolls were correctly classified to the sex from which they belong (80.0% in leave-one-out classification) (Wilks’ lambda = 0.723, χ^2^ = 10.214, df = 3, p = 0.017). Thus unambiguous sex discrimination based on the number of strokes or SSDs is not possible in the great spotted woodpecker.

**Table 3:** Differences in drumming characteristics between males and females.

Variable	Male (n = 26)	Female (n = 9)	t_33_	P-value
Number of strokes	12.0 ± 2.05	11.3 ± 2.46	0.886	0.382
SSD1 (ms)	62 ± 4.9	66 ± 3.6	-2.090	0.044
SSD2 (ms)	57 ± 3.5	61 ± 3.2	-2.937	0.006*
SSD3 (ms)	54 ± 3.3	58 ± 2.7	-2.911	0.006*
SSD4 (ms)	52 ± 3.1	55 ± 2.9	-2.926	0.006*
SSD5 (ms)	50 ± 3.2	53 ± 2.9	-2.680	0.011
SSD_min_ (ms)	40 ± 2.7	42 ± 2.7	-2.426	0.021

Results of t-tests are given. Table contains number of strokes; SSD1-5 –first five stroke-to-stroke durations, and SSD_min_−minimum stroke-to-stroke duration within a roll. Mean values ± standard deviations are given.

*—result is significant after Bonferroni correction.

### Identity coding

The analysed drumming parameters had lower within-individual than between-individual coefficients of variation, which suggests that the temporal parameters of drumming may be useful for coding identity (Table 4). Since initial predictors were strongly correlated (Table 1) we included in DFA only these predictors which mutually correlated less than 0.8 (NS, SSD1, SSD2, SSD5, SSD_min_; VIF < 5.74). Our DFA model correctly classified 69.8% of rolls to their originating individual (64.7% in leave-one-out classification) when all 41 individuals were considered together. The rate of correct classification increased when we split our dataset between males and females. For males, DFA correctly classified 74.6% (70.4% in leave-one-out classification) of rolls, while for females the result was even higher, at 88.4% (84.5% in leave-one-out classification) (Table 5). In all three DFAs, the correct classification rate was higher than the classification rate expected by chance, id est when any given call would be equally likely to be classified to any individual bird: 2.4% (1/41) for all individuals, 3.8% (1/26) for males and 11.1% (1/9) for females.

**Table 4:** Descriptive statistics of analysed characteristics of great spotted woodpecker drumming.

Variable	Mean	SD	Min	Max	CV_i_	CV_b_	PIC
Number of strokes	12.1	2.30	7.7	17.1	10.4	22.6	2.18
SSD1 (ms)	63	4.8	52	73	3.1	8.2	2.63
SSD2 (ms)	58	3.8	48	66	2.5	7.2	2.85
SSD3 (ms)	55	3.7	47	63	2.3	7.1	3.05
SSD4 (ms)	53	3.6	44	61	2.2	7.0	3.14
SSD5 (ms)	51	3.5	42	59	2.4	7.2	3.03
SSD_min_ (ms)	40	3.0	34	47	5.6	9.1	1.62

Table contains mean values of analysed drumming parameters: Number of strokes; SSD1-5 –first five stroke-to-stroke durations, SSDmin—minimum SSD. Mean values (Mean), standard deviation (SD), minimal (Min) and maximal (Max) values, within- (CV_i_) and between-individual coefficient of variation (CV_b_), and potential for identity coding (PIC) are given. Table based on 609 rolls belonging to 41 individuals.

**Table 5:** Results of three DFAs, which classify drumming of individuals, males and females.

Function	Eigenvalue	Wilks’ lambda	Percent of variance	Cumulative variance
Individuals (41 individuals, 609 rolls)				
1	10.987	0.110	55.4	55.4
2	4.503	0.022	22.7	78.1
3	2.976	0.006	15.0	93.1
4	0.830	0.0002	4.2	97.3
5	0.535	0.001	2.7	100.0
Males (26 individuals, 426 rolls)				
1	11.725	0.114	58.6	58.6
2	5.438	0.017	27.2	85.8
3	1.738	0.006	8.7	94.4
4	0.669	0.003	3.3	97.8
5	0.443	0.002	2.2	100.0
Females (9 individuals, 129 rolls)				
1	14.334	0.095	69.0	69.0
2	4.668	0.019	22.5	91.4
3	1.046	0.008	5.0	96.5
4	0.612	0.004	2.9	99.4
5	0.122	0.003	0.6	100.0

Eigenvalues, Wilks’ lambda, explanatory power are given.

**Fig 3:**
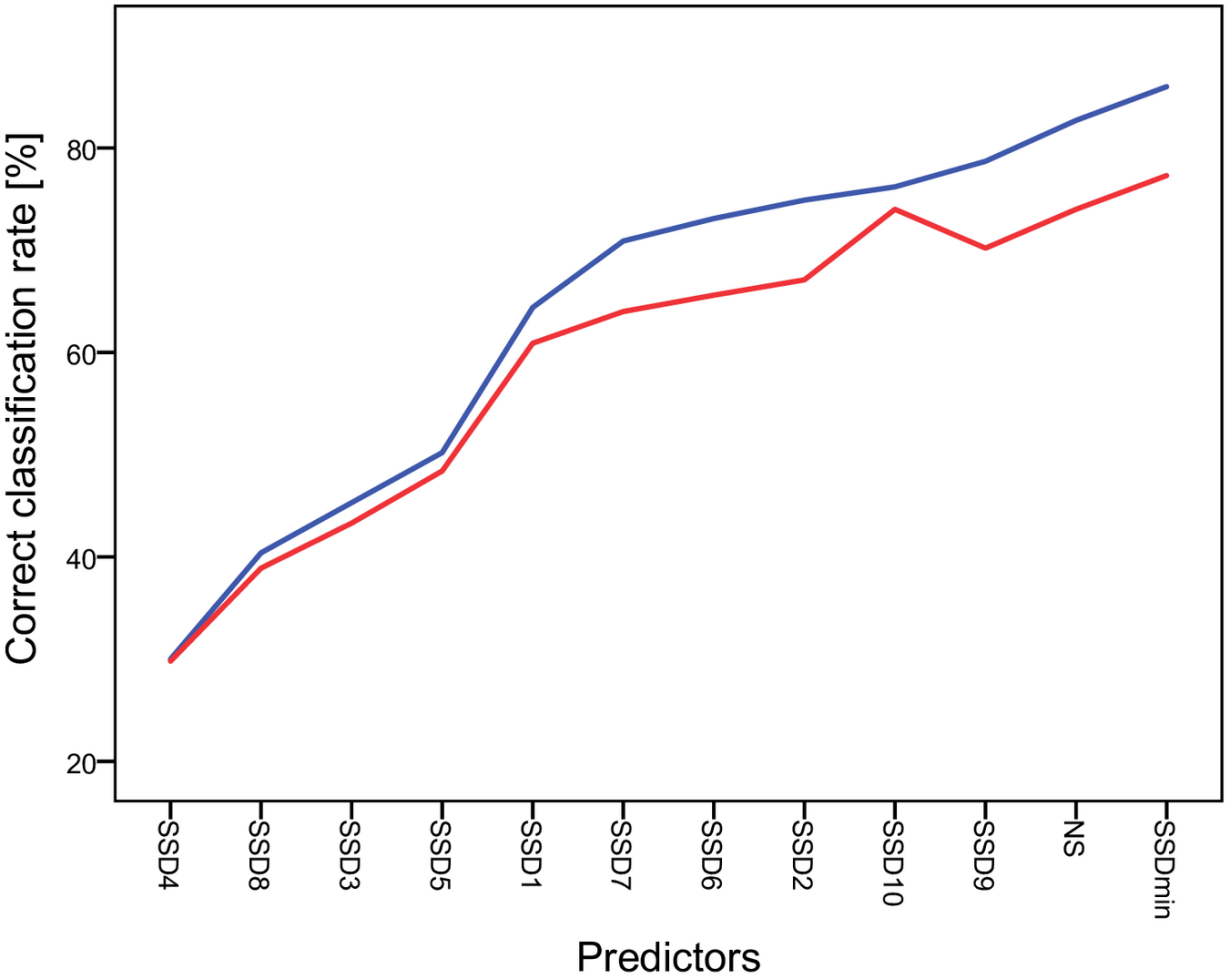
Changes in correct classification rate in models with different number of predictors. Null model contained one predictor, that with the highest PIC value. In each successive model we added the predictor with the next-highest PIC value; the final model contained 12 predictors. The correct classification rate (blue line) and correct classification rate in leave-one-out classification (red line) are given. Analysis is based on 33 individuals. NS -number of strokes; SSD1-10 –first ten stroke-to-stroke durations, SSDmin—minimum SSD.

The series of DFAs, all based on 33 individuals for which at least 11 strokes were observed in each roll, demonstrated that the rate of correct classification changed as successive predictors were added to the model. With our null model, which contained only SSD4, only 30% of classifications were correct. To this, we added, one at a time, the 12 predictors with the highest PIC value. The final model with 12 predictors correctly classified 86% of rolls, which was much higher than the rate expected by chance (3%) (Fig 3).

## Discussion

Sexual recognition and pair-synchronisation have been proposed as potential functions of great spotted woodpecker drumming [25]. Indeed, Zabka [27] suggested that, specifically, the length of drumming is important in sex recognition (females’ drumming rolls are shorter than males’). Our study could not confirm this hypothesis, since our DFA did not reveal 100% correct classification rate when we applied SSD2 or SSD2, NS, SSD_min_ to discriminate sex based on drumming. However, when we analysed each predictor separately we found that males struck faster than females—SSDs were slightly but significantly shorter in drumming produced by males than in that by females—but we still did not find a significant difference in the number of strokes within a roll. Despite the significant differences in SSD, unambiguous sex determination based on this parameter was not possible, since variation in SSD overlapped between males and females (Table 3). Thus, researchers, and probably also birds, are not able to unambiguously recognise a great spotted woodpecker’s sex based only on the number of strokes in a roll and SSD. Overlap between the acoustic parameters of male and female vocalisation has been described in a few bird species [33–36], and in the great spotted woodpecker, it is possible that drumming is merely a clue to sex determination. Instead, unambiguous sex recognition may be possible only visually, when birds can see each other, as sexual dimorphism is clearly evident in plumage patterns [25]. Alternatively, birds may use not drumming but rather calls produced by the syrinx to determine the sex of a caller via long-range acoustic communication.

The rate and duration of drumming has the potential to be an honest signal of an individual’s quality [37], since only individuals with well-developed back and neck muscles (i.e. in good condition) should be able to produce long-lasting drumming with short SSDs. On the other hand, males′ cognitive abilities, expressed as the ability to find a substrate with appropriate resonance properties (thus creating a loud sound broadcasted over a longer distance), may be also important [38]. This raises the intriguing possibility of the existence of sexual selection based on drumming. In this scenario, better-quality females (i.e. those who drum long, fast, and loudly) should choose males of better phenotypic or genetic quality as mates (i.e. those who also drum long, fast, and loudly) [39]. Therefore, we would expect that, within a pair, males should drum faster than females, but also that within-pair variation in drumming would be significantly lower than between-pair variation. Unfortunately, we lack the data to test this hypothesis.

We found that the CV_i_ of SSD and NS are lower than CV_b_ (Table 4). Thus, roll duration as well as SSD could be useful for individual recognition in the great spotted woodpecker. Our DFAs correctly classified 70–88% of rolls to an individual, depending on whether the sexes were grouped together or split into males and females. Our result is similar to those observed for call classification in other bird species, for example in African wood owls (*Strix woodfordii*) [40], European nightjars [12], corncrakes [15], or woodcocks [13]. Therefore, the temporal parameters of the great spotted woodpecker’s drumming could be useful for census and population monitoring [1]. Specifically, analyses of call recordings may help to verify whether different recordings of drumming belongs to the same or different individuals, which may be helpful in the estimation of population size. Accurate discrimination among individuals can also help in validating survey methods, in refining estimates of population size based on traditional census methods, or in determining census efficiency [13], [41].

Individual identification is more problematic than discrimination, since unambiguous identification of a particular individual across multiple observations is a much more challenging task [1]. The great spotted woodpecker is the most common woodpecker in the Western Palearctic [42]. Drumming in this species is accelerative, and the differences between the minimal and maximal value of a particular SSD are small (shorter than 20–30 ms; Fig 2). Therefore, the likelihood that a signal such as SSD could be used effectively for identity coding is low, particularly when one considers the size of the overall population (a similar situation was observed in corncrakes [43]). With this in mind, we would expect the rate of correct individual identification to decline [44] and the probability of finding two acoustically indistinguishable individuals to increase [43] with an increase in the number of individuals analysed. Therefore, we suggest that using the temporal structure of drumming to track the life history of particular individuals is rather unlikely in the great spotted woodpecker. The second limitation to the use of drumming in individual identification is that we have not recordings of drumming from the same individuals in different stages of the breeding season or in different years, as has been done for example in the corncrake [15] or the eagle owl [14]. Therefore, at this point we may only suppose that SSD remains relatively constant over an adult bird’s lifetime because is constrained by anatomy. However, also the current condition of the individual may have important influence on the SSDs.

In a biological context, individual discrimination or identification is possible when a particular acoustic feature has high potential for individual coding and receivers are able to perceive differences in the acoustic signal. With some variation depending on the species in question, birds are able to distinguish between two sounds when the intervening interval is longer than 1–4 ms [45]. Therefore, we cannot exclude the possibility that woodpeckers are able to discriminate among individuals based on timing differences at this scale. Currently, however, the functions of woodpeckers’ drumming displays are thought to be similar to those of their well-studied counterparts, passerine songs—mate attraction, territorial establishment, pair bond maintenance, and localisation of individuals [25], [27], [46]. Unfortunately, to the best of our knowledge, there is as yet no experimental evidence which would demonstrate that characteristics of drumming (rate, duration, amplitude etc.) are important in inter- and intra-sexual communication. The limited data that do exist, though, indicate that some woodpeckers species use drumming for species recognition [47]. However, it still remains unclear which characteristics of drumming are used in species recognition, and whether and how birds recognise sex or the quality of a drumming individual. The results of our study suggest that the specific temporal pattern of drumming may be important in the discrimination of nearest neighbours or mates within a pair. However, experimental studies are needed to confirm this hypothesis.

In Europe, many woodpecker species are rare and live in small, isolated populations. Some defend large-size territories throughout the year and drum from places separated by hundreds of metres, which complicates accurate estimation of population size [42]. In such species, effective determination of drumming individuality may significantly improve census accuracy. Therefore, drumming individuality in rare woodpecker species represents an interesting avenue for potential study by conservation biologists, as a tool to aid in monitoring and census tasks.

## Supporting information

S1 File An example of great spotted woodpecker drumming. Three rolls are given.
(WAV)

S1 Dataset (XLS)
